# Observation of tunable electrical bandgap in large-area twisted bilayer graphene synthesized by chemical vapor deposition

**DOI:** 10.1038/srep15285

**Published:** 2015-10-16

**Authors:** Jing-Bo Liu, Ping-Jian Li, Yuan-Fu Chen, Ze-Gao Wang, Fei Qi, Jia-Rui He, Bin-Jie Zheng, Jin-Hao Zhou, Wan-Li Zhang, Lin Gu, Yan-Rong Li

**Affiliations:** 1 State Key Laboratory of Electronic Thin Films and Integrated Devices, University of Electronic Science and Technology of China, 610054 Chengdu, China; 2 Beijing National Laboratory for Condensed Matter Physics, Institute of Physics, Chinese Academy of Sciences, Beijing, China

## Abstract

Although there are already many efforts to investigate the electronic structures of twisted bilayer graphene, a definitive conclusion has not yet been reached. In particular, it is still a controversial issue whether a tunable electrical (or transport) bandgap exists in twisted bilayer graphene film until now. Herein, for the first time, it has been demonstrated that a tunable electrical bandgap can be opened in the twisted bilayer graphene by the combination effect of twist and vertical electrical fields. In addition, we have also developed a facile chemical vapor deposition method to synthesize large-area twisted bilayer graphene by introducing decaborane as the cocatalyst for decomposing methane molecules. The growth mechanism is demonstrated to be a defined-seeding and self-limiting process. This work is expected to be beneficial to the fundamental understanding of both the growth mechanism for bilayer graphene on Cu foil and more significantly, the electronic structures of twisted bilayer graphene.

Bilayer graphene (BG) has attracted much attention due to its unique electronic structure, which can be modified by the stacking orders^1^,^2^,^3^. AA-stacked BG (AA-BG) has a massless Dirac spectrum, which results in the gapless characteristics under external electric fields similar to that of monolayer graphene (MG)^4^. In comparison, Bernal-stacked BG (AB-BG) has a massive Dirac spectrum, and a tunable bandgap can be opened by applying vertical electric fields which break the layer symmetry^5^,^6^,^7^. Thus, AB-BG with tunable bandgap is expected to be used for potential applications in digital electronics and photonics. Besides AA- and AB-BG, BG can also be stacked in the twisted sequence, where the twist angel varies from 0° to 60° (AA stacking: 0°; AB stacking: 60°)^8^,^9^,^10^. For the AB-BG growth reported previously^11^,^12^, the twisted BG regions have always observed which can apparently influence the electrical properties of AB-BG. Consequently, the investigation of electrical properties of twisted BG is not only significant for the fundamental research but also the potential applications of BG film.

Although there are several reports about the electronic structures of twisted BG, the undisputed conclusion is still lacked. Early studies suggested that twisted BG has a massless Dirac spectrum similar to that of AA-BG, and a gap cannot be opened by applying external electric fields^13^,^14^,^15^. However, in the most recent report^16^, the studies by using angle-resolved photoemission spectroscopy (ARPES) have demonstrated that the slightly twisted AA-BG (0.1°–0.4°) has the coexisting massive and massless Dirac spectra; even in the small twist angle (0.1°), a gap can be opened by the breakage of interlayer-coupling and potential symmetry, which is caused by the combination effect of twist and applied electrical fields. Therefore, it is significant to investigate whether twisted BG has a tunable electrical (or transport) bandgap under external electric fields.

In the research area of BG, another hot spot is the chemical vapor deposition (CVD) synthesis of BG film on Cu foil, which has advantages in large-area growth and low cost^17^. Until now, the CVD growths of BG film on Cu foil have been reported by many groups^18^,^19^,^20^,^21^; however, in theses previous reports, the growth conditions were more strict and complicated than those of monolayer graphene, such as long growth time (~3 h)^18^, finely tuned growth pressure^19^, and two-step process^20^,^21^. Thus, it is desirable to develop a more simple CVD method for BG growth.

Herein, a facile CVD method is proposed to synthesize the twisted BG film on Cu foil, and the growth parameters are same with those of MG film except spin-coating decaborane onto the Cu foil before growth. More significantly, the electrical studies reveal that as-synthesized twisted BG has a tunable electrical bandgap under vertical electrical fields, which has not been observed before. We expect that this work may be beneficial to the fundamental understanding of both the growth mechanism for BG film on Cu foil and more importantly, the electronic structures of twisted BG.

## Results

### Synthesis of twisted BG film

Twisted BG film was synthesized by a facile CVD method. Firstly, 25-μm-thick Cu foil (99.8%, Alfa Aesar) was cleaned in the HCl/H_2_O (1:10) solution, and then washed by DI water several times. Secondly, decaborane (B_10_H_14_) employed as the cocatalyst was spin-coated onto the Cu foil, which is critical for the formation of twisted BG film. Thirdly, the Cu foil was loaded into the silica tube of the CVD system with a vacuum background of 7 × 10^−4^ Pa, and then the growth chamber was heated to 1000 °C and held for 20 min with 30 sccm Ar, and then the CH_4_/H_2_ (15/30 sccm) replacing Ar were introduced into the tube for graphene growth at 1000 °C for 20 min. Finally, cooled the system to room temperature with a cooling rate of 50 °C /min in CH_4_/H_2_ ambience. For comparison, MG film was synthesized by the same procedure of twisted BG except without spin-coating decaborane onto the Cu foil.

### Transmission electron microscopy characterization of twisted BG film

**Figure 1:**
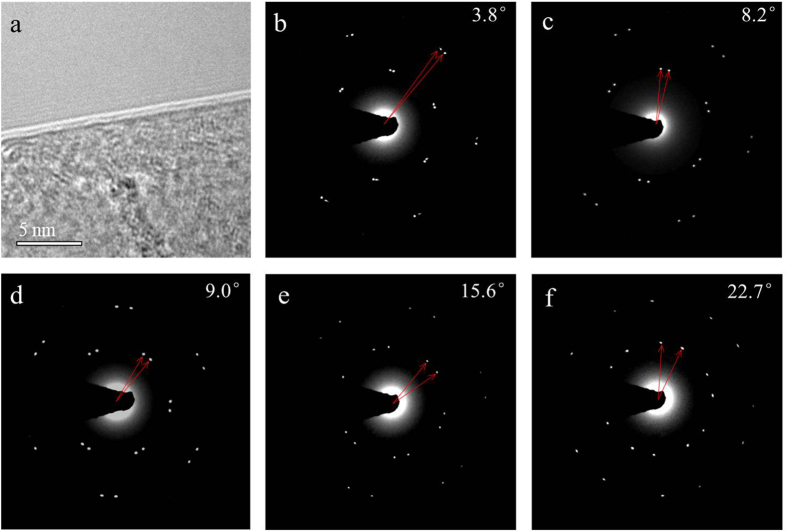
TEM characterization of twisted BG film. (**a**) High-resolution TEM image of the random edge of twisted BG film on a TEM grid. (**b**–**f**) SAED patterns of the twisted BG film taken from 5 random regions. The corresponding rotation angles are 3.8°, 8.2°, 9.0°, 15.6° and 22.7°, respectively.

Transmission electron microscopy (TEM) was carried out to characterize the structure of the as-synthesized twisted BG film. Figure 1a shows the high-resolution TEM image of random edge of graphene film, indicating bilayer structure. 8 random selected area electron diffraction (SAED) patterns have been obtained, and all reveal two typical hexagonal crystalline structure of graphene with rotation angles. As shown in Fig. 1b–f and Supplementary Fig. S1, the rotation angle varies from 0 to 30° in different regions (3.8°, 8.2°, 9.0°, 9.2°, 15.6°, 16.6°, 18.9° and 22.7°), which indicates that the twisted BG film has polycrystalline structure with varied twisted stacking orders^11^. It is noted that neither AA- nor AB- stacking orders are observed. It means even if there are some AA-BG or AB-BG regions in the twisted BG, the proportion would be very small.

### Optical characterizations of twisted BG films

**Figure 2:**
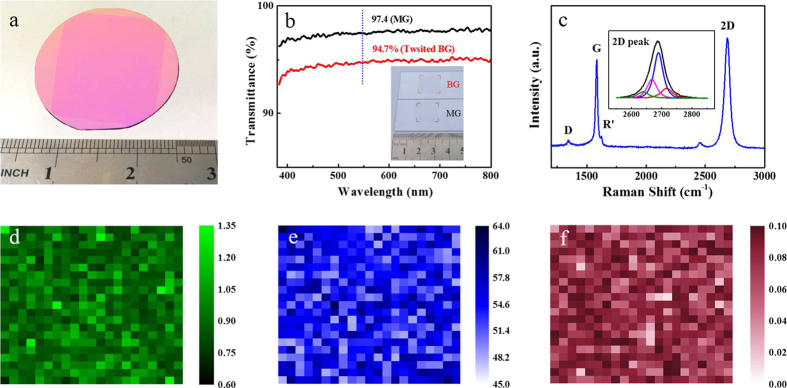
Optical characterization of twisted BG films. (**a**) Photograph of large-area twisted BG film transferred onto a 285-nm SiO_2_/Si substrate with a size of 1 inch × 1 inch. (**b**) Transmittances of MG and twisted BG films transferred onto quartz substrates. The inset shows the photograph of MG and twisted BG films transferred onto quartz substrates. (**c**) Typical Raman spectrum of twisted BG film transferred onto a 285-nm SiO_2_/Si substrate; the inset shows that the 2D peak can be deconvoluted into four peaks. (**d**) *I*_G_/*I*_2D_, (**e**) FWHM of 2D peak and (**f**) *I*_D_/*I*_G_ Raman mappings of twisted BG film at the 100 × 100 *μ*m^2^ scale.

Figure 2a shows the photograph of large-area twisted BG film transferred onto a 285-nm SiO_2_/Si substrate with a size of 1 inch × 1 inch, which shows high uniformity. The thickness of twisted BG film was further confirmed by measuring the optical transmittances. As shown in Fig. 2b, at 550 nm, the twisted BG film transferred onto a quartz substrate shown in the inset of Fig. 2b has a transmittance of 94.7%, which is 2.7% smaller than that of MG (97.4%). This result is good agreement with that of BG films reported previously^19^,^22^.

Raman spectroscopy was used to further investigate the structure, uniformity and quality of twisted BG film. Figure 2c shows the typical Raman spectrum of twisted BG film transferred onto a 285-nm SiO_2_/Si substrate substrate^23^. The G to 2D peak intensity ratio (*I*_G_/*I*_2D_) is ~0.8 and the full-width at half-height maximum (FWHM) of the 2D peak is ~52 cm^−1^, indicating bilayer graphene^11^,^20^,^24^. Additionally, the asymmetric 2D peak shown in the inset of Fig. 2c can be deconvoluted into four peaks, corresponding to the four permissible photon transition processes in characteristic bilayer graphene^24^. Figure 2d,e show the Raman mapping of *I*_G_/*I*_2D_ and 2D peak FWHM at the 100 × 100 *μ*m^2^ scale, respectively. Based on the previous reports^11^, the bilayer regions can be confirmed by the *I*_G_/*I*_2D_ ratio from 0.7 to 1.3 and the FWHM value of 2D peak from 45 to 60 cm^−1^. Thus, from the data shown in Fig. 2d,e, the coverage of twisted BG can be estimated to be ~98%, indicating its high uniformity; the other 2% regions correspond to few-layer graphene. Further, the Raman mapping of the D to G peak intensity ratio (*I*_D_/*I*_G_) is shown in Fig. 2f, and it reveals that all values of *I*_D_/*I*_G_ are below 0.1, suggesting the high quality of twisted BG film^11^. In addition, we also notice that the values of 2D FWHM and *I*_G_/*I*_2D_ intensity ratio of our twisted BG are higher than those for MG and similar to those for AB-BG^17^,^18^,^19^,^20^. The broadened 2D band and enhanced intensity ratio of *I*_G_/*I*_2D_ can be attributed to two reasons: the twist angle^25^,^26^,^27^,^28^ and disorder^21^ for our twisted BG. The detailed discussion can be seen in the Supplementary Information (Page 2–3).

In order to further investigate the distribution of twist angles in the twisted BG film, 50 random regions have been studied by Raman spectra in the area within 1 × 1 cm^2^. 26 out of 50 regions have the R’ Raman bands (~1622 cm^−1^; see Fig. 2c or the green spectrum in Supplementary Fig. S2a), indicating that the range of twist angle is 5° ~ 10° 25,26,27,28. 6 out of 50 regions have the R Raman bands (~1489 cm^−1^; see the blue spectrum in Supplementary Fig. S2a), indicating that the range of twist angle is 10° ~ 14° 25,26,27,28. Furthermore, according to the FWHM and intensity of 2D band shown in Supplementary Fig. S2b and Fig. S2c, the histogram of twist angles of 50 regions can be finally determined^25^,^26^,^27^,^28^. As shown in Supplementary Fig. S2d, the proportion of twisted angles smaller than 10° is 74%, indicating that grains with small twist angle (<10°) are predominant in the polycrystalline twisted BG film.

## Discussion

**Figure 3:**
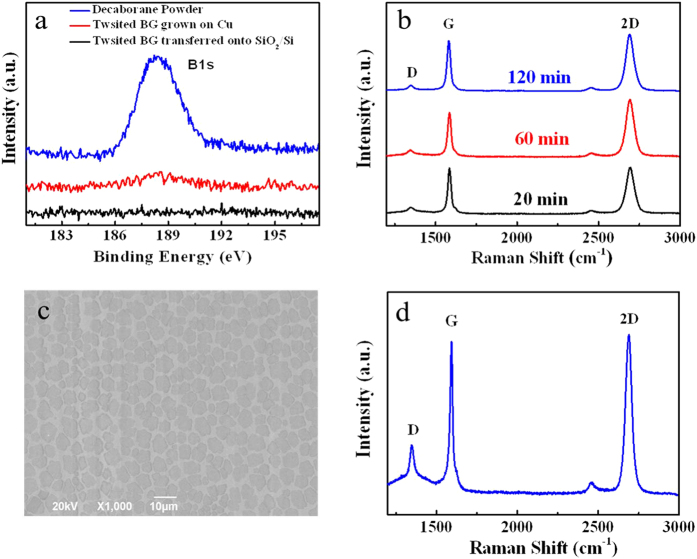
Investigation of growth mechanism of twisted BG film. (**a**) XPS B1s spectra of decaborane powder, twisted BG film grown on Cu foil and transferred onto a 285-nm SiO_2_/Si substrate. (**b**) Typical Raman spectra of twisted BG films grown for 20, 60 and 120 min growth. (**c**) SEM image of BG islands on the Cu foil synthesized for 5 min growth. (**c**) Typical Raman spectrum of twisted BG islands synthesized for 5 min growth.

In the growth process of twisted BG film, decaborane is believed to be as the cocatalyst for promoting the decomposition of methane molecules on the Cu foil, which can be demonstrated as follows. Firstly, for comparative studying, the graphene film was synthesized by using the same procedure of twisted BG except without spin-coating decaborane onto the Cu foil before growth. The high-resolution TEM and Raman spectrum shown in Supplementary Fig. S3a and Fig. S3b reveal that the as-synthesized graphene film is monolayered^29^. It suggests that decaborane is a most critical factor for growing twisted BG film. Furthermore, the effect of decaborane was studied by performing X-ray photoelectron spectroscopy (XPS) measurements. As shown in the Fig. 3a, there is a weak B peak in the high-resolution B1s XPS spectrum of twisted BG film grown on Cu foil; however, the position of B peak is the same as that of decaborane, suggesting that this weak B peak is originated from the residual decaborane on the twisted BG film. Moreover, after transferring the twisted BG film onto a 285-nm SiO_2_/Si substrate, any B-related XPS signal is below the detection limit, which implies that residual decaborane can be effectively removed during transferring process. Therefore, the above studies have confirmed that decaborane do not react with graphene film or Cu foil, and only simply plays the cocatalytic role of decomposing methane molecules on the Cu foil during the growth process of twisted BG^30^.

For further understanding the growth mechanism of twisted BG, the effect of growth time on the graphene thickness was investigated. When the growth time is 20 min, the twisted BG film is continuous and fully covers the Cu foil. When the growth time is increased to 120 min, the thickness of graphene film remains unchanged which can be confirmed by the Raman spectra shown in Fig. 3b^12^. Furthermore, the initial stage of growth was studied by setting the growth time at 5 min. As shown in the scanning electron microscopy (SEM) image of Fig. 3c, graphene islands are formed on Cu foil for 5 min growth. From the corresponding Raman spectrum of graphene islands shown in Fig. 3d, the *I*_G_/*I*_2D_ intensity ratio and 2D band FWHM value are ~1.0 and ~50 cm^−1^, respectively, indicating bilayer graphene^19^. It is noted that 10 random islands have been studied by Raman spectra, and all regions show bilayer structures. Thus, we can conclude that the growth of twisted BG is not a layer-by-layer mechanism, and its thickness is defined at the initial stage of growth.

**Figure 4:**
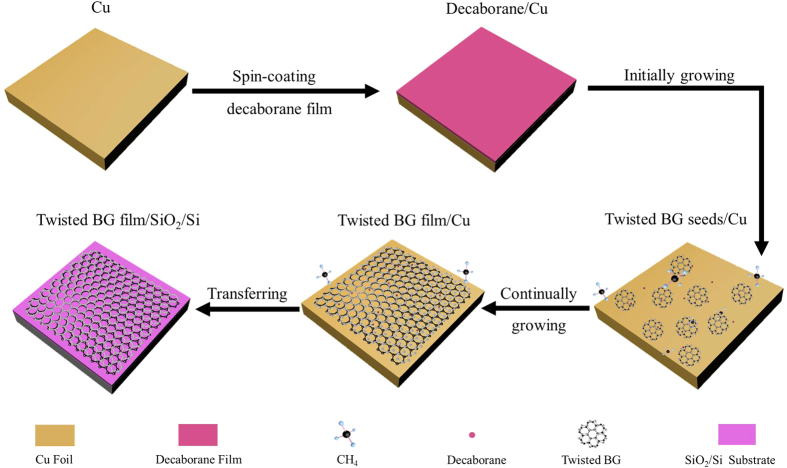
Schematic illustration of proposed growth mechanism of twisted BG film synthesized on the Cu foil. Firstly, the Cu foil was spin-coated with decaborane film; then the twisted BG seeds were formed on the Cu foil at the initial growth stage; then the twisted BG seeds merge into a continuous film with increasing the growth time; finally, the twisted BG film was transferred onto the SiO_2_/Si substrate.

Figure 4 shows the schematic illustration of proposed growth mechanism of twisted BG film synthesized on the Cu foil. At the initial growth stage, methane molecules are catalytically decomposed on the surface of Cu foil with the assistance of decaborane, and subsequently BG islands (seeds) are formed. When the growth time is further increased, BG islands laterally grow with the maintenance of their thickness. Finally, these islands merge into a continuous BG film. This mechanism is most likely a defined-seeding and self-limiting process, which is similar with that of BG film synthesized under a finely tuned growth pressure^19^. It is noted that our twisted BG film is formed under the similar growth conditions of monolayer graphene, which is due to the cocatalytic effect of decaborane. It implies that the multilayer graphene may be formed by adopting the more effective cocatalyzer for prompting methane decomposition. In addition, AB stacking region has not been observed in this work. Pervious report indicated that high surface diffusion coefficient was beneficial for the formation of AB stacking order with lowest energy configuration^20^, thus we think that besides being as the cocatalyzer for methane decomposition, decaborane also hinders the diffusion of active carbon species, resulting in the formation of both varied twisted stacking orders and disorder for the twisted BG film^20^,^31^.

**Figure 5:**
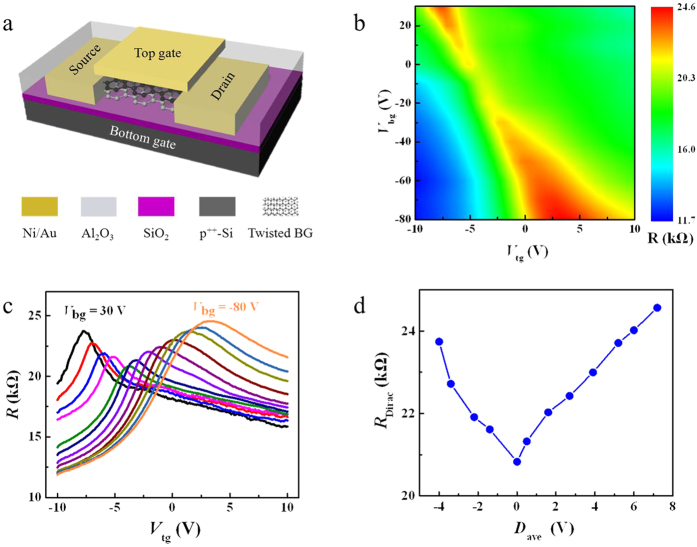
Electrical properties of twisted BG film. (**a**) Schematic illustration of twisted BG FET with dual-gate. (**b**) Two-dimensional contour plot of the device resistance *R* versus *V*_tg_ and *V*_bg_. (**c**) Curves of *R* as a function of *V*_tg_ at fixed *V*_bg_ ranging from −80 to 30 V, with 10 V increment. (**d**) Variation of the Dirac resistance, *R*_Dirac_, as a function of the average displacement field, *D*_ave_.

Electrical transport measurements haven been performed to investigate the electronic structures of twisted BG film, in particular to verify the existence of tunable electrical bandgap. Figure 5a shows the schematic structure of dual–gated BG field effect transistor (FET). The BG channel is sandwiched completely between top and bottoms gates; 70 nm Al_2_O_3_ and 285 nm SiO_2_ act as top-gate and bottom-gate dielectric films, respectively. The detailed fabrication procedures are given in the Methods Section. For this dual-gated structure, the electrical bandgap and carrier doping concentration of twisted BG can be independently controlled by the effects of top and bottom gate voltages (*V*_tg_ and *V*_bg_), which can be described by the top and bottom electrical displacement fields (*D*_t_ and *D*_b_). *D*_t_ and *D*_b_ can be calculated using the following equations


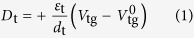


and


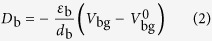


respectively. *ε*_*t*_ and *d*_*t*_ are dielectric constant and thickness of top-gate dielectric layer, respectively 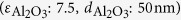; *ε*_*b*_ and *d*_*b*_ are dielectric constant and thickness of bottom-gate dielectric layer, respectively 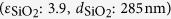; 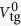 and 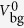 are the Dirac offset top and bottom voltages due to the initial environment-induced carrier doping, respectively^5^.

Figure 5b shows a two-dimensional contour plot of the device resistance *R* versus *V*_tg_ and *V*_bg_. The values of *R* reach maximum at the upper-left and lower-right corners, where average displacement fields (*D*_ave_) are highest. *D*_ave_ is defined as


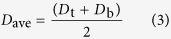


The variation of *R* is similar with that of AB-BG, which implies a tunable bandgap opening in twisted BG under vertical electric fields^5^,^20^,^32^. This can be more evidently illustrated in the curves of *R* as a function of *V*_tg_ at different *V*_bg_ shown in Fig. 5c. *V*_tg_ is swept from −10 V to 10 V at different fixed *V*_bg_ ranging from −80 V to 30 V as steps of 10 V. For each curve, the Dirac resistance *R*_Dirac_ has the highest value, corresponding to the charge neutrality (*D*_t_ = *D*_b_). As shown in Fig. 5c, *R*_Dirac_ increases with increasing *V*_bg_ in both positive and negative directions, which implies that the bandgap increases with increasing the electrical displacement fields^5^,^20^. Furthermore, because the lower bandgap causes the lower *R*_Dirac_, the Dirac point with the lowest *R*_Dirac_ can be identified as the zero-gap Dirac point (*D*_t_ = *D*_b_ = 0) and used to estimate the Dirac offset voltage induced by the environmental doping 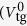 = −3.7 V, 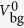 = −10 V). Consequently, the relationship of *R*_Dirac_ versus *D*_ave_ can be obtained as shown in Fig. 5d. It shows that the *R*_Dirac_ reaches the maximum value at the highest *D*_ave_, which further confirms the tunability of bandgap in twisted BG film^17^,^20^,^32^.

**Figure 6:**
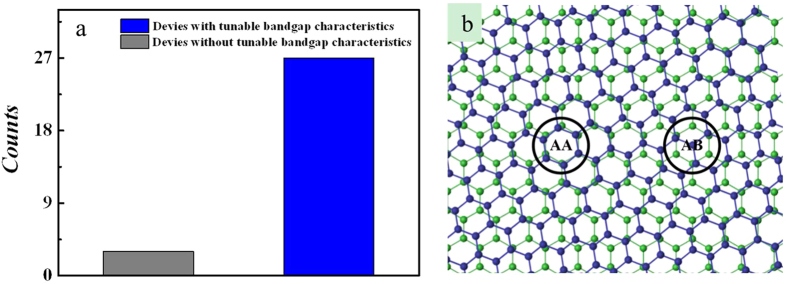
Statistical analysis of electrical properties of devices and schematic illustration of twisted BG. (**a**) Histogram of tunable electrical bandgap characteristics for dual-gated BG FET devices. 27 out of 30 devices show the tunable electrical bandgap characteristics. (**b**) A twisted BG for a rotation angle of 9.0°. AA and AB regions are shown.

30 dual-gated BG FET devices have been measured, and moreover, for comparison, the dual-gated MG FETs have also been fabricated and measured. As shown in Fig. 6a, the electrical studies reveal that 27 out of 30 dual-gated BG FET devices show the tunable electrical bandgap characteristics; the other 3 devices show the typical zero-gap characteristic, which is similar with that for the dual-gated MG FET shown in Supplementary Fig. S4^32^. Thus, based on the TEM and electrical studies, we can conclude that a tunable electrical bandgap is indeed opened in twisted BG under the combination effect of twist and vertical electrical fields. To our best knowledge, it is the first time to observe the electrical bandgap with tunable characteristics for twisted BG film.

Furthermore, it is noted that previous report indicated that for CVD polycrystalline graphene, the mean grain size was smaller than 2 μm^33^; for our polycrystalline twisted BG, the grain size may be further decreased because besides being as the cocatalyzer for methane decomposition, decaborane also hinders the diffusion of active carbon species. Thus, we can conclude that the device channel for dual-gated BG FET (4 μm × 10 μm) contains several grains of different twisted stacking orders. It is known that the interlayer coupling increases with decreasing rotation angle for twisted BG; especially, as shown in Fig. 6b, when the rotation angle is small (<10°), the AA and AB regions (similar with AA and AB stacking BG) emerge, corresponding to strong interlayer coupling^14^. Consequently, we think that the tunable electrical bandgap characteristics are originated from grains with small rotation angles (<10°), which are predominant in our polycrystalline twisted BG film shown in Supplementary Fig. S2d. Furthermore, our electrical result and previous APRES study^16^ both imply that there is a transition of the tunable bandgap with twist angle. Although the exact effect mechanism of twist angle on the electronic structures of twist BG film need to be further investigated in future studies, we think the first observation of tunable electrical bandgap for twisted BG is beneficial for the fundamental understanding of its electronic structures. In addition, the carrier mobilities of twisted BG film extracted form 30 fabricated devices can be estimated to be 672–1695 cm^2^/V·s by using the well-developed procedures (see the Methods Section)^20^,^34^, which can be compared to those of CVD BG films reported previously^17^,^21^.

In summary, the twisted BG film has been synthesized on the Cu foil by a facile CVD method by introducing decaborane as the cocatalyst for decomposing methane molecules. Furthermore, the electrical studies reveal that the combination effect of twist and vertical electrical fields can break the interlayer-coupling and potential symmetry, and open a tunable electrical bandgap in twisted BG film. This work is beneficial to the fundamental understanding of not only the growth mechanism for BG film, but also the electronic structures of twisted BG.

## Methods

### CVD growth of twisted BG film

Initially, 25-μm-thick Cu foil (99.8%, Alfa Aesar) was cleaned in the dilute HCl/H_2_O (1:10) solution for 3 min, and then washed by deionization (DI) water several times to remove the residual acid solution, and then was dried by nitrogen gas. Secondly, decaborane was dissolved into anisole solvent (0.03 g/ml), and then spin-coated onto the surface of Cu foil at 3000 rpm for 30 s. Thirdly, the Cu foil was loaded into the silica tube of the CVD system with a vacuum background of 7 × 10^−4^ Pa, and then the growth chamber was heated to 1000 °C and held for 20 min with 30 sccm Ar, and then the CH_4_/H_2_ (15/30 sccm) replacing Ar was introduced into the tube for graphene growth at 1000 °C for 20 min. Finally, cooled the system to room temperature with a cooling rate of 50 °C /min in CH_4_/H_2_ ambience.

### CVD growth of MG film

Monolayer graphene film was synthesized under the same growth parameters of twisted BG except without spin-coating decaborane onto the Cu foil before growth.

### Transfer process of graphene films

Firstly, poly(methyl methacrylate) solution (PMMA, A4) was spin-coated on the surface of graphene grown on the Cu foil at 3000 rpm for 30 s, and then the Cu foil was etched away in the FeCl_3_ solution for 12 h. Secondly, PMMA/graphene film was rinsed repeatedly using the DI water (10 times), and then transferred to the H_2_O/HCl/H_2_O_2_ (20:1:1) solution for 15 min for removing the residual Cu particles, and then transferred to the H_2_O/NH_4_OH/H_2_O_2_ (20:1:1) solution for 15 min for removing the insoluble organic contaminants. Note that the PMMA/graphene film was rinsed using the DI water to remove the residual solution after each cleaning step. Thirdly, the PMMA/graphene was transferred onto the target substrate (SiO_2_/Si or quartz substrates), and then was cured at 150 °C for 10 min after natural drying, and then the PMMA was removed using acetone^35^.

### Characterization

The high-resolution TEM images and SAED pattern were taken with an FEI Tecnai G2 microscope. The optical transmittances were measured by using a Perkin-Elmer model Lambda 750 UV–vis–NIR spectrophotometer. The Raman spectra were collected with a Renishaw InVia Raman microscope using a 514-nm laser beam (20 mW; 1 cm^−1^). XPS was performed on a Kratos XSAM800 using Al Ka radiation (144 W, 12 mA, 12 kV).

### Fabrication of graphene devices and Electrical measurement

Dual-gate graphene FET devices were fabricated as follows. Firstly, graphene film (twisted BG or MG) was transferred onto a 285 nm SiO_2_/Si substrates. Secondly, source and drain electrodes (Ni/Au: 50/50 nm) were defined and deposited by using photolithography and e-beam evaporation (285 nm SiO_2_ and *p*^++^-Si acted as the bottom-gate dielectric and electrode, respectively). Thirdly, the graphene was patterned into strips (4 μm width and 10 μm length) as device channels by using photolithography and Oxygen plasma etching. Fourthly, 50-nm-thick Al_2_O_3_ top-gate dielectric film was deposited as the top gate dielectric by atomic layer deposition (ALD). Fifthly, top-gate electrodes were patterned and then metals were evaporated (Ni/Au: 50/50 nm). Finally, the devices were annealed at 250 °C for 2 h with H_2_/Ar (100/100 sccm).

Electrical measurements were carried out with an Agilent 4155B semiconductor parameter analyzer in air at room temperature. To extract the field-effect mobility of graphene FET devices, the total resistance of the device, *R*_total_, can be expressed as below^34^:


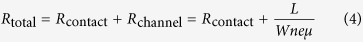


where *R*_contact_ is the contact resistance of the metal/graphene contact; *R*_channel_ is the resistance of graphene channel; *L* and W are the channel and width length, respectively; *n* is the carrier concentrations in the graphene channel region; *e* is the electron charge; *μ* is carrier mobility. *n* can be approximated by


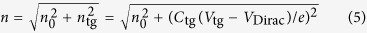


where *n*_0_ represents the density of carriers at Dirac point; *n*_tg_ is top-gate-modulated carrier density; *C*_tg_ is the top-gate capacitance 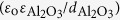
36. In order to extract the carrier mobility, we define


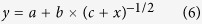


where 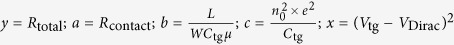 to fit the measured data. So the carrier mobility can be obtained by


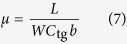


## Additional Information

**How to cite this article**: Liu, J.-B. *et al.* Observation of tunable electrical bandgap in large-area twisted bilayer graphene synthesized by chemical vapor deposition. *Sci. Rep.* **5**, 15285; doi: 10.1038/srep15285 (2015).

## Supplementary Material

Supplementary Information
