# Supplementary material for: Observation of tunable electrical bandgap in large-area twisted bilayer graphene synthesized by chemical vapor deposition
Source: Sci Rep. 2015 Oct 16;5:15285. doi: 10.1038/srep15285 (PMC4607884; doi:10.1038/srep15285)
Supplement: Supplementary Information [file srep15285-s1.pdf]

SUPPLEMENTARY INFORMATION

Observation of tunable electrical bandgap in large-area twisted bilayer graphene synthesized by chemical vapor deposition

Jing-Bo Liu[1]*, Ping-Jian Li[1]*, Yuan-Fu Chen[1], Ze-Gao Wang[1], Fei Qi[1], Jia-Rui He[1], Bin-Jie Zheng[1], Jin-Hao Zhou[1], Wan-Li Zhang[1], Lin Gu[2] & Yan-Rong Li[1]

[1]State Key Laboratory of Electronic Thin Films and Integrated Devices, University of Electronic Science and Technology of China, 610054 Chengdu, China. [2]Beijing National Laboratory for Condensed Matter Physics, Institute of Physics, Chinese Academy of Sciences, Beijing, China *These authors contributed equally to this work. Correspondence and requests for materials should be addressed to P.J.L. (email: lipingjian@uestc.edu.cn) or Y.F.C. (email: yfchen@uestc.edu.cn)

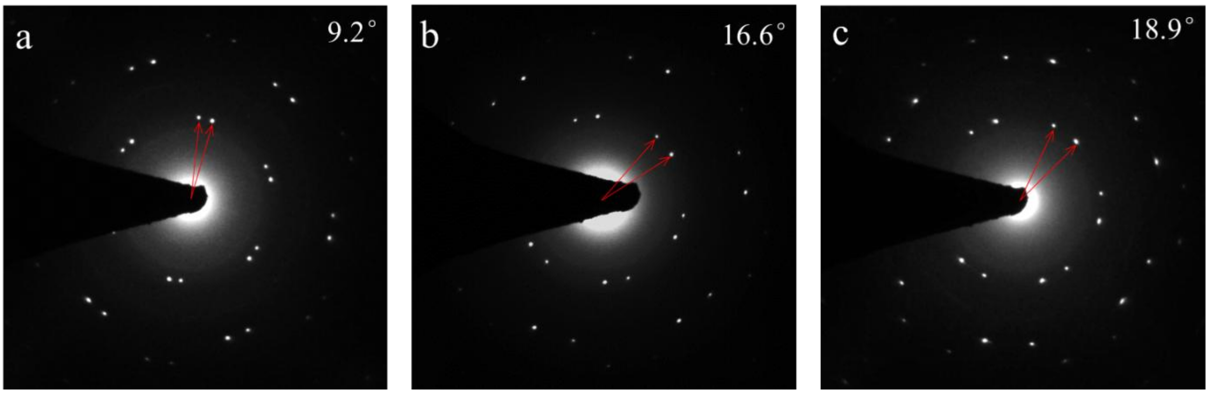

Supplementary Figure S1. TEM characterizations of twisted bilayer graphene (BG). (**a-c**) SAED patterns of the twisted BG film taken from 3 random regions. The corresponding rotation angles are 9.2^{o}, 16.6^{o} and 18.9^{o}, respectively.

Raman 2D band and I_G/I_{2D} intensity ratio

For our twisted BG film, the values of 2D FWHM and I_G/I_{2D} intensity ratio are higher than that for MG and similar to that for AB-BG[1-3]. The broadened 2D band and enhanced intensity ratio of I_G/I_{2D} can be attributed to two reasons: the twist angle[4-7] and disorder[8] for our twisted BG. The detailed discussion is as follows.

(1) The effects of broadening 2D band and enhancing I_G/I_{2D} intensity ratio are partly ascribed to the twist angle. Previous Raman studies[4-7] revealed that when the rotation angle was smaller than 10°, the values of 2D FWHM and I_G/I_{2D} intensity ratio were higher than that for MG and close to that for AB-BG. It means that for the regions with small rotation angles in twisted BG, the 2D band and I_G/I_{2D} intensity ratio can be broadened and enhanced, respectively.

It is noted that TEM studies (new Fig. 1 and Supplementary Fig. S1) reveal that the rotation angle varies from 0 to 30° in different regions for our twisted BG, and only 4 out of 8 random SAED patters show rotation angles smaller than 10°. On the other hand, we do not observe the Raman values similar with that for MG. Thus, there is another reason for the broadened Raman 2D band and enhanced intensity ratio of I_G/I_{2D} (especially for the regions with large rotation angles), which is discussed below.

(2) Another reason is the disorder induced during growth process in our twisted BG:

① Yan et al. reported the observation of broadening effect of Raman 2D band of AB-BG, which can be due to the disorder induced during the growth process[8].

② The features of broadened 2D band and enhanced intensity ratio of I_G/I_{2D} in this work were also observed in the other twisted BG reported previously[9-11]. We notice that this work and refs. 9-11 have one thing in common: the growth methods can cause more disorder than that for AB-BG reported previously[1-3]. On the one hand, for this work, decaborane coated onto copper foil before growth hinders the diffusion of active carbon species, which causes the formation of not only varied twisted stacking orders (rotation angle: 0 to 30°) but also disorder for the twisted BG; for refs. 9-11, the twisted BG films have been formed between the Ni[9,10] (or Cu[11]) layer and the SiO_2 substrate, where the space limit effect hinders the diffusion of active carbon species, resulting the formation of not only varied twisted stacking orders (rotation angle: 0 to 30°) but also disorder. Whereas, on the other hand, for AB-BG reported previously[1-3], active carbon species diffuse onto clean Cu surface without disturbance. The high surface diffusion coefficient results the formation of AB stacking order with the low energy state and high-quality film with little disorder.

In summary, we think that both the twist angle and disorder contribute to the broadened 2D band and enhanced intensity ratio of I_G/I_{2D} for our twisted BG.

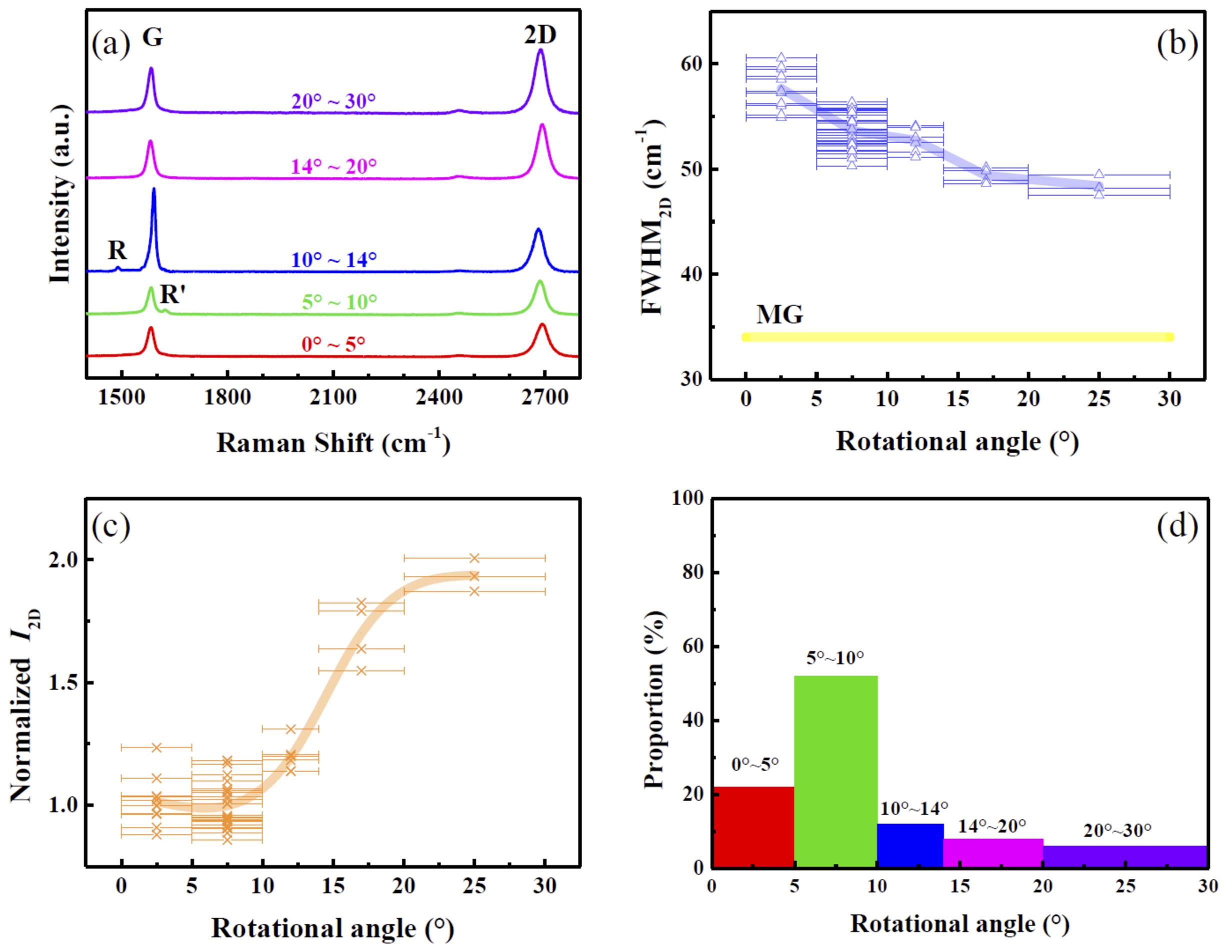

Supplementary Figure S2. Distribution of twist angels in the polycrystalline twisted BG film determined by Raman spectra. (**a**) Raman spectra from twisted BG regions with different twist angle. (**b**) FWHM of the 2D band. **(c)** Intensity of the 2D band (normalized to the MG value). **(d)** Histogram of twisting angles of 50 regions in our polycrystalline twisted BG film.

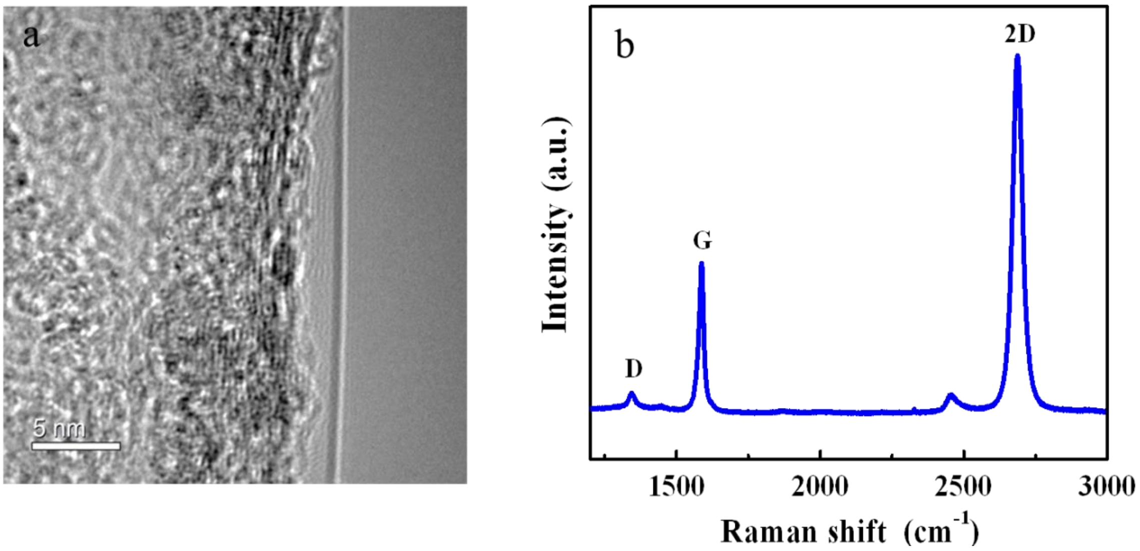

Supplementary Figure S3. TEM and Raman characterizations of monolayer graphene (MG) film synthesized under the same growth parameter of twisted BG except without spin-coating decaborane onto the Cu foil. (**a**) High-resolution TEM image of as-synthesized MG film. (**b**) Typical Raman spectrum of as-synthesized MG film. The values of I_G/I_{2D} and 2D peak FWHM are ~0.4 and ~ 40 cm^{-1}, indicating monolayer structure.

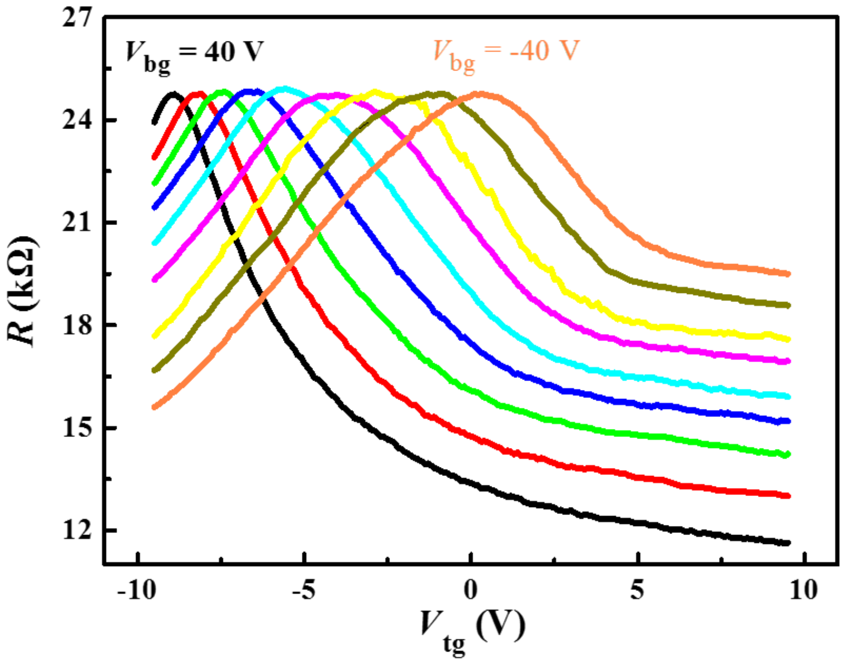

Supplementary Figure S4. Electrical properties of monolayer graphene FET with dual-gate. Curves of device resistance R as a function of V_{tg} at fixed V_{bg} ranging from -40 to 40 V, with 10 V increment. V_{tg} and V_{bg} are top and bottom gate voltages, respectively. The values of R_{Dirac} almost remain unchanged with varying V_{bg}, indicating the typical zero-gap characteristics of monolayer graphene[12].

Supplementary References

1. Lee, S., Lee, K. and Zhong, Z. Wafer scale homogeneous bilayer graphene films by chemical vapor deposition. *Nano Lett.* **10,** 4702-4707 (2010).
2. Luo, Z. et al. Large scale synthesis of bi-layer graphene in strongly coupled stacking order. *Adv. Funct. Mater.* **21,** 911-917 (2010).
3. Liu, L. et al. High-yield chemical vapor deposition growth of high-quality large-area AB-stacked bilayer graphene. *ACS Nano* **6,** 8241-8249 (2012).

4 Kim, K. et al. Raman spectroscopy study of rotated double-layer graphene: misorientation-angle dependence

of electronic structure. *Phys. Rev. Lett.* **108**, 246103 (2012).

5 Carozo, V. et al. Raman signature of graphene superlattices. *Nano Lett.* **11**, 4527-4534 (2011).

6 Havener, R. W., Zhuang, H., Brown, L., Hennig, R. G., and Park J. Angle-resolved Raman imaging of interlayer rotations and interactions in twisted bilayer graphene. *Nano Lett.* **12**, 3162-3167 (2012).

7 He, R. et al. Observation of low energy Raman modes in twisted bilayer graphene. *Nano Lett.* **13**, 3594-3601 (2013).

8. Yan, K., Peng, H., Zhou, Y., Li, H. and Liu, Z. Formation of bilayer bernal graphene: layer-by-layer epitaxy via chemical vapor deposition. *Nano Lett.* **11,** 1106-1110 (2011).

9. Yan, Z. et al. Growth of bilayer graphene on insulating substrates. *ACS Nano* **5,** 8187-8192 (2011).

10. Peng, Z., Yan, Z., Sun, Z. and Tour, J. M. Direct growth of bilayer graphene on SiO_2 substrates by carbon diffusion through nickel. *ACS nano* **5,** 8241-8247 (2011).

11. Su, C. Y. et al. Direct formation of wafer scale graphene thin layers on insulating substrates by chemical vapor deposition. *Nano Lett.* **11,** 3612-3616 (2011).

12 Xia, F., Farmer, D. B., Lin, Y. and Avouris, P. Graphene field-effect transistors with high on/off current ratio and large transport band gap at room temperature. *Nano Lett.* **10,** 715-718 (2010).
